# Cost Analysis of Universal Screening vs. Risk Factor-Based Screening for Methicillin-Resistant *Staphylococcus aureus* (MRSA)

**DOI:** 10.1371/journal.pone.0159667

**Published:** 2016-07-27

**Authors:** Virginia R. Roth, Tara Longpre, Doug Coyle, Kathryn N. Suh, Monica Taljaard, Katherine A. Muldoon, Karamchand Ramotar, Alan Forster

**Affiliations:** 1 Department of Medicine, The Ottawa Hospital and University of Ottawa, Ottawa, Ontario, Canada; 2 Ottawa Hospital Research Institute, Ottawa, Ontario, Canada; 3 School of Epidemiology, Public Health and Preventive Medicine, University of Ottawa, Ottawa, Ontario, Canada; 4 Health Economics Research Group, Brunel University, Uxbridge, Middlesex, United Kingdom; 5 Department of Pathology and Laboratory Medicine, The Ottawa Hospital and University of Ottawa, Ottawa, Ontario, Canada; Kliniken der Stadt Köln gGmbH, GERMANY

## Abstract

**Background:**

The literature remains conflicted regarding the most effective way to screen for MRSA. This study was designed to assess costs associated with universal versus risk factor-based screening for the reduction of nosocomial MRSA transmission.

**Methods:**

The study was conducted at The Ottawa Hospital, a large multi-centre tertiary care facility with approximately 47,000 admissions annually. From January 2006-December 2007, patients underwent risk factor-based screening for MRSA on admission. From January 2008 to August 2009 universal MRSA screening was implemented. A comparison of costs incurred during risk factor-based screening and universal screening was conducted. The model incorporated probabilities relating to the likelihood of being tested and the results of polymerase chain reaction (PCR) testing with associated effects in terms of MRSA bacteremia and true positive and negative test results. Inputted costs included laboratory testing, contact precautions and infection control, private room costs, housekeeping, and length of hospital stay. Deterministic sensitivity analyses were conducted.

**Results:**

The risk factor-based MRSA screening program screened approximately 30% of admitted patients and cost the hospital over $780 000 annually. The universal screening program screened approximately 83% of admitted patients and cost over $1.94 million dollars, representing an excess cost of $1.16 million per year. The estimated additional cost per patient screened was $17.76.

**Conclusion:**

This analysis demonstrated that a universal MRSA screening program was costly from a hospital perspective and was previously known to not be clinically effective at reducing MRSA transmission. These results may be useful to inform future model-based economic analyses of MRSA interventions.

## Introduction

Methicillin-resistant *Staphylococcus aureus* (MRSA) is a pathogen of increasing concern and is associated with higher hospital readmission rates, poorer prognosis, and increased mortality resulting in increasing costs to the healthcare system [1]. Infection control interventions play a critical role in preventing the transmission of MRSA within the healthcare environment [2,3]. While infections due to MRSA can cause significant morbidity and mortality [4,5], the majority of patients are asymptomatic carriers who can serve as silent reservoirs for further transmission [6]. Furthermore, MRSA colonization can persist for months to years [6]. Admission screening for MRSA using rapid detection methods has been recommended to facilitate timely detection of asymptomatic carriers and implementation of infection control measures [7–9].

Admission screening can involve screening all patients at the time of admission to hospital (universal screening) [8], or selectively screening patients with certain high-risk factors for MRSA (risk factor-based screening) [10–12]. There is conflicting evidence in the literature regarding which approach is most effective in reducing nosocomial MRSA transmission [13–15]. While several studies have assessed the clinical efficacy of universal versus risk factor-based screening programs for MRSA [16], few studies have assessed the cost effectiveness [17,18]. Studies suggests that risk factor-based screening is clinically effective in reducing the transmission of MRSA within the hospital setting and can be a cost effective strategy [3,10–12,19–23]. Studies in our institution have found that the introduction of a universal MRSA screening program did not significantly affect the rates of nosocomial MRSA compared to risk factor-based screening [24].

The objective of this analysis was to compare the annual and per patient costs of a universal MRSA screening intervention compared to risk factor-based screening.

## Methods

### Study design and setting

The study was conducted at The Ottawa Hospital, a large multi-site tertiary care facility with inpatient beds on three separate campuses. Infection control practices are standard across all campuses. There are approximately 47 000 admissions per year and approximately 1 200 beds for medical, surgical, obstetrical, critical care, mental health and rehabilitation patients [25].

From January 2006 to December 2007 (24 months) patients were screened on admission for MRSA using a risk factor-based approach. From January 2008 to August 2009 (20 months) universal MRSA screening designed to screen all admitted patients (excluding newborns) was implemented. A detailed explanation of the screening protocol has been previously published [24] and analyses determined that universal MRSA screening was not clinically effective at reducing the rates of nosocomial MRSA compared to risk-factor based screening in this setting.

In this analysis, a cost comparison was conducted comparing a universal screening program and a risk factor-based screening program. The analysis incorporated: 1) operating costs to the hospital to implement the universal screening program, including laboratory costs for testing and costs for specimen collection; 2) costs of managing additional MRSA cases identified by the universal MRSA screening intervention (costs associated with infection control, contact precautions, housekeeping, private room); and 3) assessing costs of fewer nosocomial cases (healthcare costs associated with MRSA colonization and infection).

### Laboratory and infection control methods

Screening specimens were obtained from the nares and rectum of each patient, as well as any open skin lesions (up to a maximum of two sites) and catheter exit sites, where applicable. Up to three swabs per patient were pooled and inoculated into a single tube of selective broth consisting of brain heart infusion broth (Becton Dickinson, Sparks, MD) supplemented with 5 μg/ml of aztreonam (ICN, Costa Mesa, CA) and 75 μg/ml of ceftizoxime (GlaxoSmithKline, Inc.) and incubated at 35°C overnight [26]. Screening for MRSA by the IDI-MRSA assay was performed by transferring a 50-μl aliquot of the overnight broth into the sample reagent buffer provided with the assay kit (GenOhm). The PCR was then performed according to the manufacturer’s instructions by using a SmartCycler II device (Cepheid, Sunnyvale, CA) as previously described [27]. Results were generally available within 24 hours of specimen collection. In our setting, the PCR assay has a negative predictive value of 98% but a positive predictive value of only 65%; therefore, PCR-positive broth samples underwent culture confirmation [28].

PCR-positive broths were inoculated onto MRSA*Select*^™^ plates (Bio-Rad Laboratories, Quebec, Canada) and incubated in O_2_ at 35°C for 24 hours. Pink colonies growing on the MRSA*Select*^™^ plates were confirmed as MRSA using latex slide or tube coagulase and and detection of the modified penicillin binding protein (PBP 2a) by latex agglutination (Oxoid Inc.) as previously described [27].

Patients with a positive PCR test were placed on contact precautions until hospital discharge or documented eradication of MRSA. Patients who tested PCR-positive but whose culture results were negative were considered to be false-positives and had their contact precautions discontinued. At the time of this study, routine MRSA decolonization was not performed. Patients found to be colonized or infected with MRSA while in a multi-bed room were moved to a private room, and the other beds were blocked to new admissions until the roommates’ screening results were available.

### Decision model

**Fig 1:**
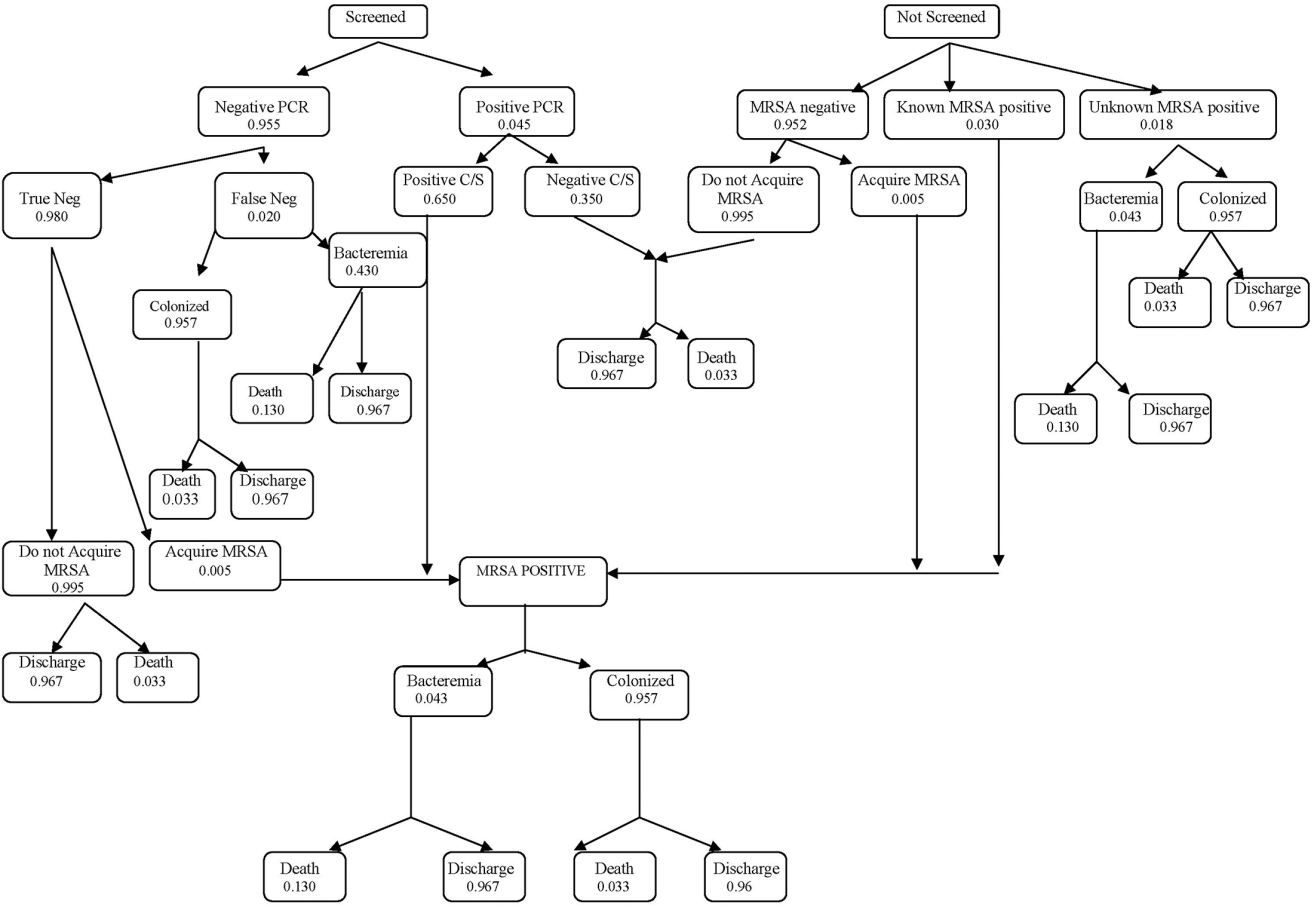
Schematic of decision tree.

A balanced and symmetrical decision tree model was built by identifying the health states that defined each clinical scenario leading to MRSA bacteremia or colonization (Fig 1) [29]. Each node in the model was associated with relevant probabilities of events and assigned costs. Data for the health states and probability estimates are presented in Table 1 and a detailed breakdown of the associated health costs of MRSA screen are presented in Table 2. The model was structured in Microsoft Excel XP (2002).

**Table 1:** Health states and probability estimates for MRSA screening.

Chance node	Probability	Probability estimate	Source
Screening	Probability screened versus not screened for MRSA within 48h of admission under risk factor based screening	0.292	Ottawa hospital warehouse data
	Probability screened versus not screened for MRSA within 48h of admission under universal screening	0.838	Ottawa hospital warehouse data
PCR Result	Probability admission screen for MRSA is PCR positive versus negative	0.045	Ottawa hospital warehouse data
Result of culture	Probability PCR positive specimen is MRSA culture negative (false positive PCR) versus culture positive (true positive PCR)	0.350	Ottawa hospital warehouse data
Results when unscreened	Probability not screened on admission and MRSA negative	0.952	Conterno et al.[27]
	Probability not screened on admission and MRSA positive	0.030	
	Probability not screened on admission and MRSA status unknown	0.018	
True negative	Probability patient is MRSA negative based on negative predictive value of PCR test (true negative PCR)	0.980	Conterno et al. [27]
MRSA acquisition in hospital	Remains MRSA negative during hospital admission versus acquires MRSA during hospital stay	0.995	Ottawa hospital warehouse data
MRSA bacteremia	Probability MRSA positive has MRSA bacteremia versus colonized with MRSA	0.043	Ottawa hospital warehouse data
Final Outcome	Probability patient with MRSA bacteremia dies while in hospital versus discharged	0.130	Coello et al.[30]
	Probability patient without MRSA bacteremia dies while in hospital versus discharged	0.033	Van Walraven et al.[29]

**Table 2:** Healthcare costs associated with MRSA screening, colonization and bacteremia.

Service	Cost (CAD)*	Source
**1. Laboratory costs**		
MRSA PCR costs		
Supply cost	$17.68	Hospital finance data
Labour cost	$2.24	Hospital finance data
Total	$19.92	Hospital finance data
MRSA Culture costs		
Positive culture	$16.59	Hospital finance data
Negative culture	$3.93	Hospital finance data
PCR positive/culture positive	$36.51	Hospital finance data
PCR positive/culture negative	$23.85	Hospital finance data
**2. Contact precautions and infection control**		
Estimated for 50 contacts/patient/day		Conterno et al.[26]**
Material costs		
Gowns @ $0.50 each	$25.00	Conterno et al.[26]
Gloves @ $0.15 each	$7.50	Conterno et al.[26]
Masks @ $0.13 each	$6.50	Conterno et al.[26]
Human resource costs		
Time to put on gowns, gloves and mask (1 min)		Conterno et al.[26]
Cost of nursing time/patient/day	$29.15	Conterno et al.[26]
Total contact precautions cost /day	$68.15	
Cost of infection control profession/hour	$41.07	Hospital finance data
Time/new case of MRSA (30 min)	$20.54	Conterno et al.[26]
Time/false positive and known MRSA case (15 min)	$10.27	Conterno et al.[26]
**3. Private room**		
Loss revenue due to private room use (cost/bed)	$220.00	Hospital finance data
Loss revenue due to blocked bed (per diem cost/room)	$180.00	Hospital finance data
**4. Housekeeping**		
Routine room cleaning	$42.62	Conterno et al.[26]
Isolation room cleaning	$66.69	Conterno et al.[26]
Difference (incurred when patient moved from multi-bed to private room when found to be MRSA positive)	$24.07	Conterno et al.[26]
**5. Length of stay**		
Medical/surgical stay costs/patient/day	$1219	Hospital finance data

*Costs adjusted from 2005 to 2010 rates using Bank of Canada Inflation calculator

**Analyses by Conterno et al. use data from The Ottawa Hospital

A patient’s admission to hospital and whether or not they were screened for MRSA was the first step in the model (Fig 1). At each chance node, individual patients had the probability of competing events allowing them to move through the decision tree. Probabilities related to screening, the results of PCR, probability of MRSA bacteremia and final outcomes relating to death or discharge were calculated. Of the distinct chance nodes in the model, six were populated from the actual dataset of 147,975 admissions sourced from The Ottawa Hospital Data Warehouse during the study period [24], three were populated from previous analyses conducted by our study team on our in-patient population [28,30], and one was derived from a published study with a comparable in-patient population [31]. For the purposes of this analysis, we defined “infection” as having MRSA bacteremia. Please see web-appendix (S1 Table: Health states and probability estimates for MRSA screening) for a detailed description of the health states and assigned probabilities with reference to the nodes in Fig 1.

The model was applied to each screening period to compare the accumulated costs for a patient screened upon admission during the risk factor-based screening period (where 29.2% of patients were screened upon admission) and the universal screening period (where 83.8% of admissions were screened) [24].

### Cost analysis

The healthcare costs associated with MRSA detection included increases in length of hospital stay (LOS), laboratory testing, contact precautions, housekeeping, and lost revenue attributed to private room use or blocked beds. Cost data were sourced directly from the hospital finance systems or from previous work by our research team and presented in Table 2 and include costs associated with laboratory tests, contact precaution and infection control, private room, housekeeping and length of stay [28]. Human resource costs associated with contact precautions and infection control were restricted to tasks deemed to be directly related to MRSA including putting on gowns, gloves and masks. Additional costs were considered to be covered by general nursing salary and were consistent between both screening periods. The only exception was that estimates associated with increased length of stay attributed to MRSA bacteremia were derived from a study conducted in a comparable Canadian hospital, reporting that the average patient length of stay was 7 days compared to an average length of stay for MRSA infected patients of 24 days [32]. Thus, for the purposes of this model, 17 additional hospital days were attributed to each patient with MRSA bacteremia to account for additional costs of the infection. We assumed no increase in length of stay associated with MRSA colonization. Therefore, for colonized patients, we used the average length of stay for our in-patient population of 8 days. All costs and data sources are presented in Table 2.

### Sensitivity analysis

Deterministic sensitivity analysis was performed to evaluate the potential for additional costs to be incurred in the risk-factor screening period that are not captured in the model. Specifically, little was known about the costs associated with: 1) patients unscreened for MRSA and therefore not known to carry MRSA, and 2) patients with false-negative screening tests. For instance, an ‘unscreened’ patient who was unknowingly MRSA positive upon admission may not have incurred costs in the original model as we were unaware of their status. However, upon suspicion of an infection, this patient might subsequently have a clinical culture taken during the hospital stay deeming them MRSA infected and thus incurring costs. The same might be true for a false negative patient who may, in fact, be MRSA infected and have additional costs attributed to care. As these costs were not documented or easily supported by the literature, they could not be included in the original model. Therefore, sensitivity analysis examined the effect that these potential added costs would have on the universal MRSA screening intervention.

Further deterministic analyses were conducted in order to test the robustness of the findings, and probabilities were altered across all chance nodes in the model. All data were de-identified by the data warehouse prior to access and analysis. Strict protocols were followed to ensure privacy and confidentiality for all patients. Ethics approval was obtained from The Ottawa Hospital Research Ethics Board [ID: 2008620-01H].

## Results

A total of 147,975 admissions sourced from The Ottawa Hospital Data Warehouse were used. Table 3 presents the per patient costs associated with MRSA detection and control measures, and the comparative total costs accrued during each screening period. Patients previously known to have MRSA incurred costs during subsequent admissions. The additional costs per re-admission were $1,604 for patients with an MRSA bacteremia, and $385 for patients colonized with MRSA. Input costs attributed to newly identified patients were $1,834 for an MRSA bacteremia, and $599 for MRSA colonization. The cost associated with a false positive screening test was $526 per patient.

**Table 3:** Costs (per patient) associated with risk factor-based compared to universal MRSA screening.

	Cost (CAD)	Risk factor-based screening (24 months)	Universal screening (20 months)	Comments
**Previously known MRSA—bacteremia**		**n = 8**	**n = 14**	
Laboratory cost	$19.92	$149.90	$280.10	Cost of PCR test
Contact precautions /day	$68.15	$8,718.09	$16,290.37	Average 17 days
Infection control time	$10.27	$77.28	$144.41	For known MRSA case
Private room /day	$220.00	$28,143.50	$52,588.14	Average 17 days
Housekeeping	$66.69	$501.84	$937.73	Isolation room cleaned on discharge
Length of stay cost /day	$1,219.00	$155,940.58	$291,386.10	Average 17 days
**Total**	**$1,604.03**	**$193,531.19**	**$361,626.84**	
**Previously known MRSA—colonized**		**n = 167**	**n = 313**	
Laboratory cost	$19.92	$3,336.10	$6,233.74	Cost of PCR test
Contact precautions /day	$68.15	$91,307.37	$170,614.34	Average 8 days
Infection control time	$10.27	$1,719.97	$3,213.88	For known MRSA case
Private room /day	$220.00	$294,756.00	$550,772.64	Average 8 days
Housekeeping	$66.69	$11,168.91	$20,869.00	Isolation room cleaned on discharge
Length of stay cost /day	$0	$0	$0	No associated length of stay costs
**Total**	**$385.03**	**$402.288.35**	**$751,704.51**	
**Newly MRSA bacteremia**		**n = 6**	**n = 12**	
Laboratory cost	$36.51	$207.30	$428.59	PCR positive/culture positive
Contact precautions /day	$68.15	$6,578.19	$13,600.22	Average 17 days
Infection control time	$20.54	$116.63	$241.12	For new MRSA case
Private room /day	$220.00	$21,235.53	$43,903.86	Average 17 days
Lost revenue due to blocked bed	$180.00	$1,022.03	$2,113.02	Assumed 1 day per new case
Housekeeping	$66.69	$378.66	$782.87	Isolation room cleaned on discharge
Housekeeping cost difference	$24.07	$136.67	$282.56	Isolation clean to move patient to private room
Length of stay cost /day	$1,219.00	$117,664.16	$243,267.30	Average 17 days
**Total**	**$1,834.96**	**$147,339.17**	**$304,619.54**	
**Newly MRSA colonized**		**n = 126**	**n = 261**	
Laboratory cost	$19.92	$2,517.24	$5,204.32	Cost of PCR test
Contact precautions /day	$68.15	$68,895.51	$142,439.50	Average 8 days
Infection control time	$20.54	$2,595.59	$5,366.30	For new MRSA case
Private room /day	$220.00	$222,406.63	$459.819.36	Average 8 days
Lost revenue due to blocked bed	$180.00	$22,746.13	$47,026.98	Assumed 1 day per new case
Housekeeping	$66.69	$8,427.44	$17,423.50	Isolation room cleaned on discharge
Housekeeping cost difference	$24.07	$3,041.66	$6,288.55	Isolation clean to move patient to private room
Length of stay cost /day	$0	$0	$0	No associated length of stay costs
**Total**	**$599.37**	**$330,630.20**	**$683,568.51**	
**False Positive for MRSA**		**n = 71**	**n = 147**	
Laboratory cost	$23.85	$1,693.35	$3505.95	PCR positive culture negative
Contact precautions /day	$68.15	$9,677.30	$20,036.10	For 48 hours until culture confirmed negative
Infection control time	$10.27	$729.17	$1,509.69	For false positive case
Private room /day	$220.00	$31,240.00	$64,680.00	For 48 hours until culture confirmed negative
Lost revenue due to blocked beds	$180.00	$12,780.00	$26,460.00	Assumed 1 day per new case
Housekeeping cost difference	$24.07	$1,708.97	$3,538.29	Isolation clean to move patient to private room
**Total**	**$526.34**	**$57,828.79**	**$119,730.03**	
**All other screens**		**n = 21,893**	**n = 51,068**	
Laboratory cost	$19.92	$436,108.56	$1,017,274.56	Cost of PCR incurred with negative screens
**Total**	**$19.92**	**$436,108.56**	**$1,017,274.56**	
**Overall Total**		**$1,567,726.25**	**$3,238,523.99**	

Costs associated with risk factor-based screening compared to universal screening are presented in Table 4. In total, the risk factor-based screening (24 months) cost the hospital $1.57 million and the universal screening (20 months) cost the hospital $3.24 million, resulting in an additional cost of $1.67 million. Scaled annually, risk factor-based screening cost the hospital $783 773/year compared to $1 942 892/year during the universal screening period, an additional cost of $1.16 million/year to implement an universal MRSA screening intervention.

**Table 4:** Actual and estimated costs associated with risk factor-based compared to universal MRSA Screening.

		Actual costs			Estimated annual costs		
Costs		Risk factor-based screening costs (24 months)	Universal screening costs (20 months)	Cost difference	Risk factor-based screening annual costs	Universal screening annual costs	Annual cost difference
1. Laboratory	Total	$444 012.45	$1 032 927.26	-$588 914.81	$222 006.23	$619 756.32	-$397 750.09
	Bacteremia	$357.20	$708.69	-$351.49	$178.60	$425.21	-$246.61
	Colonized	$5 853.34	$11 438.06	-$5 584.72	$2 926.67	$6 862.84	-$3 936.17
2. Contact precautions (including infection control costs)	Total	$185 176.46	$362 980.53	-$177 804.07	$92 498.77	$217 566.05	-$125 067.28
	Bacteremia	$15 296.28	$29 890.59	-$14 594.31	$7 648.14	$17 934.35	-$10 286.21
	Colonized	$160 202.88	$313 053.84	-$152 850.96	$80 101.44	$187 832.30	-$107 730.86
3. Housekeeping	Total	$25 364.16	$50 123.40	-$24 759.24	$12 682.08	$30 074.04	-$17 391.96
	Bacteremia	$1 017.17	$2 003.16	-$985.99	$508.59	$1 201.90	-$693.31
	Colonized	$22 638.01	$44 581.95	-$21 943.94	$11 319.01	$26 749.17	-$15 430.16
4. Private room	Total	$597 781.66	$1 171 764.00	-$573 982.34	$298 890.83	$703 058.40	-$404 167.57
	Bacteremia	$49 379.03	$96 492.00	-$47 112.97	$24 895.52	$57 895.20	-$32 999.68
	Colonized	$517 162.63	$1 010 592.00	-$493 429.37	$258 581. 32	$606 355.20	-$347 773.88
5. Length of stay	Total	$273 604.74	$534 653.40	-$261 048.66	$136 802.37	$320 792.04	-$183 989.67
	Bacteremia	$273 604.74	$534 653.40	-$261 048.66	$136 802.37	$320 792.04	-$183 989.67
	Colonized	$0.00	$0.00	$0.00	$0.00	$0.00	$0.00
Overall*	**Total**	**$1 567 726.25**	**$3 238 523.99**	**-$1 670 797.74**	**$783 773.67**	**$ 1 942 892.13**	**-$1 159 118.46**
	**Bacteremia**	$405,346.90	$792,133.59	-$386 786.69	$202 673.45	$475 280.15	-$272 606.70
	**Colonized**	$705,856.86	$1,379,665.85	-$673 808.94	$352 928.43	$827 799.48	-$474 871.05

*Costs adjusted from 2005 to 2010 rates using Bank of Canada Inflation Calculator

The greatest increase in costs attributed to universal screening included the laboratory costs ($397 750/year) and revenue loss due to private room use or blocked beds ($404 167/year) (Table 4). As expected, the laboratory costs were greater in the universal screening period as more screening tests were conducted. Finally, additional costs were attributed to increased length of stay ($183 989/year), contact precautions ($125 067/year), and housekeeping ($17 391/year) as more patients with MRSA colonization and bacteremia were identified during the universal screening period (Tables 3 and 4).

Table 5 displays the costs associated with each clinical scenario and health state calculated in the model presented in Fig 1. It was estimated that the per patient cost during the risk-factor based screening period was $128.03 compared to $145.79 during the universal screening period, for an additional cost of $17.76 per person screened.

**Table 5:** Cost of patient care and associated probabilities for each clinical scenario and health state.

State	Cost / pt	Risk factor-based screening probability	Universal screening probability	Risk factor-based screening cost	Universal screening cost	Reference to model
Screened for MRSA–PCR neg.–True neg.–No acquisition–Discharged	$19.92	0.261142165	0.756411788	$5.20	$15.07	P12
Screened for MRSA–PCR neg.–True neg.–No acquisition—Death	$19.92	0.00891178	0.025813432	$0.18	$0.51	P13
Screened for MRSA–PCR neg.—False neg.—Bacteremia—Death	$19.92	3.0963E-05	8.9686E-05	$0.00	$0.00	P16
Screened for MRSA–PCR neg.—False neg.—Bacteremia—Discharge	$19.92	0.000207214	0.000600206	$0.00	$0.01	P17
Screened for MRSA–PCR neg.—False neg.—Colonized—Death	$19.92	0.000174927	0.000506686	$0.00	$0.01	P18
Screened for MRSA–PCR neg.—False neg.—Colonized—Discharge	$19.92	0.005125896	0.014847422	$0.10	$0.30	P19
Screened for MRSA—PCR pos.–Culture neg.—Discharge	$811.97	0.004416773	0.01279341	$3.59	$10.39	P22
Screened for MRSA—PCR pos.–Culture neg.—Death	$811.97	0.000150728	0.00043659	$0.12	$0.35	P23
Not screened for MRSA–MRSA neg.—No acquisition—Discharge	$0.00	0.650073312	0.146495394	$0.00	$0.00	P22
Not screened for MRSA–MRSA neg.—No acquisition—Death	$0.00	0.022184508	0.004999326	$0.00	$0.00	P23
Not screened for MRSA—Unknown MRSA status—Bacteremia—Death	$0.00	7.30278E-05	1.6457E-05	$0.00	$0.00	P28
Not screened for MRSA—Unknown MRSA status—Bacteremia—Discharge	$0.00	0.000488724	0.000110135	$0.00	$0.00	P29
Not screened for MRSA—Unknown MRSA status—Colonized—Death	$0.00	0.000412574	9.29745E-05	$0.00	$0.00	P30
Not screened for MRSA—Unknown MRSA status—Colonized—Discharge	$0.00	0.012089674	0.002724434	$0.00	$0.00	P31
Screened for MRSA–PCR neg.—True neg.—Acquire MRSA—Bacteremia—Death	$25,949.36	7.58594E-06	2.19731E-05	$0.20	$0.57	P34
Screened for MRSA–PCR neg.- True neg.—Acquire MRSA—Bacteremia—Discharge	$25,949.36	5.07674E-05	0.00014705	$1.32	$3.82	P35
Screened for MRSA–PCR neg.—True neg.—Acquire MRSA—Colonized—Death	$2,616.42	4.28572E-05	0.000124138	$0.11	$0.32	P36
Screened for MRSA–PCR neg.—True neg.—Acquire MRSA—Colonized—Discharge	$2,616.42	0.001255844	0.003637618	$3.29	$9.52	P37
Screened for MRSA–PCR pos.–Culture pos.—Bacteremia—Death	$25,819.46	4.74172E-05	0.000137346	$1.22	$3.55	P34
Screened for MRSA–PCR pos.–Culture pos.—Bacteremia—Discharge	$25,819.46	0.00031733	0.000919164	$8.19	$23.73	P35
Screened for MRSA–PCR pos.–Culture pos.—Colonized—Death	$2,494.26	0.000267886	0.000775945	$0.67	$1.94	P36
Screened for MRSA–PCR pos.–Culture pos.—Colonized–Discharged	$2,494.26	0.007849867	0.022737545	$19.58	$56.71	P37
Not screened for MRSA- MRSA neg.—Acquire MRSA- Bacteremia—Death	$25,929.44	1.8884E-05	4.25556E-06	$0.49	$0.11	P34
Not screened for MRSA–MRSA neg.—Acquire MRSA—Bacteremia—Discharge	$25,929.44	0.000126378	2.84795E-05	$3.28	$0.74	P35
Not screened for MRSA–MRSA neg.—Acquire MRSA—Colonized—Death	$2,596.50	0.000106686	2.4042E-05	$0.28	$0.06	P36
Not screened for MRSA–MRSA neg.—Acquire MRSA—Colonized—Discharge	$2,596.50	0.003126232	0.000704503	$8.12	$1.83	P37
Not screened for MRSA—Known MRSA pos.—Bacteremia—Death	$25,698.51	0.000119067	0.000026832	$3.06	$0.69	P34
Not screened for MRSA—Known MRSA pos.—Bacteremia—Discharge	$25,698.51	0.000796833	0.000179568	$20.48	$4.61	P35
Not screened for MRSA—Known MRSA pos.—Colonized—Death	$2,382.16	0.00067078	0.000152536	$1.60	$0.36	P36
Not screened for MRSA—Known MRSA pos.—Colonized—Discharge	$2,382.16	0.019711425	0.004442011	$46.96	$10.58	P37
**TOTAL**		**1**	**1**	**$128.03**	**$145.79**	**(-$17.76)**

Pt = patient, neg. = negative, pos = positive

### Sensitivity analysis

A sensitivity analysis was conducted to determine if various factors might alter the costs associated with the universal MRSA screening intervention compared to risk factor-based screening (Table 6). The probability of a patient being MRSA negative on admission was the most sensitive input parameter affecting costs. In our study population, if 97–99% of all patients were PCR negative on admission, the hospital could save between $3.03 and $30.75 per patient screened with universal screening compared with risk factor-based screening. Conversely, if only 90% of patients were PCR negative, the additional cost of the universal screening would increase considerably to $93.98 per patient screened. Changing the estimated probability of a true negative from a base value of 98% to between 90–99% had a minimal impact on cost, and the universal screening program was consistently more expensive ($17.00–17.85 per patient screened). As the probability of positive culture confirmation increases to 80%, the cost increases from $17.76 per screened patient to $27.72 per screened patient. As there were no additional costs attributed to death, the probability of death did not alter the cost of the risk factor-based screening compared to universal screening. In addition, the probability of MRSA acquisition and percentage of patients with MRSA bacteremia (versus MRSA colonization) did not dramatically alter costs according to this sensitivity analysis. For other probabilities relating to the probability of being a true negative, the probability of death, the probability of MRSA acquisition and percentage of patients with MRSA bacteremia (versus MRSA colonization) did not dramatically alter costs according to this sensitivity analysis. Interestingly, only when acquisition rates are very high (>60%) does universal admission screening become less costly then risk factor-based screening at a savings of $0.74 saved per patient screened.

**Table 6:** Sensitivity analysis results per patient screened.

Scenario		Cost per patient		
	Revised value	Risk factor-based screening	Universal screening	Incremental cost of risk factor-based screening
Base case		$128.03	$145.79	-$17.76
Revised percentage screened on admission under universal screening (base 84%)	75%	$128.03	$142.88	-$14.85
	95%	$128.03	$149.34	-$21.31
Revised probability that PCR is negative (base 96%)	90%	$168.22	$262.20	-$93.98
	92%	$153.61	$219.87	-$66.26
	94%	$138.99	$177.54	-$38.54
	97%	$117.07	$114.04	$3.03
	98%	$109.76	$92.87	$16.89
	99%	$102.46	$71.70	$30.75
Revised probability that a negative PCR is a true negative (base 98%)	90%	$127.63	$144.63	-$17.00
	95%	$127.88	$145.35	-$17.47
	99%	$128.08	$145.93	-$17.85
Revised probability that culture confirms positive PCR (base 65%)	40%	$119.27	$120.41	-$1.14
	50%	$122.78	$130.56	-$7.79
	60%	$126.28	$140.71	-$14.43
	70%	$129.78	$150.86	-$21.08
	80%	$133.29	$161.01	-$27.72
Revised probability of acquiring MRSA in hospital 0.5%	0.25%	$119.51	$137.34	-$17.83
	1%	$145.08	$162.68	-$17.60
	5%	$281.45	$297.80	-$16.36
	10%	$451.91	$466.71	-$14.80
Revised probability of in-hospital death with MRSA bacteremia (base 13%)	6%	$128.03	$145.79	-$17.76
	20%	$128.03	$145.79	-$17.76
Revised probability of in-hospital death without MRSA bacteremia (base 3.3%)	1.6%	$128.03	$145.79	-$17.76
	5%	$128.03	$145.79	-$17.76
Revised probability of MRSA bacteremia on admission (base 4.3%)	2%	$109.52	$127.51	-$18.00
	3%	$117.57	$135.46	-$17.89
	6%	$141.72	$159.29	-$17.57
	8%	$157.82	$175.18	-$17.37
	15%	$214.17	$230.80	-$16.63

The sensitivity analyses included costs of ‘false negative’ and ‘unknown’ ‘unscreened’ MRSA infected cases into the model. The incorporation of these cases would alter the incremental cost per patient from universal screening from $17.76 to $18.18 per patient. Please see web-appendix (S2 Table: Sensitivity analysis: Cost of patient care and associated probabilities for each clinical scenario and health state including ‘false negatives’ and ‘unknown’ MRSA bacteremia cases) for a complete table of the adjusted calculations. Please see the web-appendix for all data and calculations (S1 Dataset: MRSA costing analysis publicly available dataset).

## Discussion

To our knowledge, this is one of the first large-scale studies using actual hospital data, rather than data derived from the literature, to examine the costs associated with a hospital-wide universal MRSA admission screening intervention compared to a risk factor-based program. This analysis found that a universal screening program cost $17.76 more per patient compared to risk factor-based screening.

Economic analyses of universal screening programs to reduce nosocomial MRSA infection have shown varying results depending on the patient population, unit under analysis, and existing prevalence of MRSA [33]. Kang et al. (2012) found that compared to no screening, risk factor-based screening had lower costs and prevented more MRSA nosocomial infections, and universal screening identified more MRSA infections but was more costly. However, this study was based on estimates derived from the literature [34]. A study among surgical populations found that universal admission screening for MRSA reduced the risk of MRSA infection compared with risk-factor based screening and pre-emptive isolation, but was not cost effective in the setting of low MRSA prevalence and good infection control practices [35]. This study and others [36] suggest that universal screening may be more cost effective with higher MRSA prevalence. Conversely, we found that costs increased dramatically with higher MRSA prevalence since universal screening was not associated with a reduction in MRSA transmission at our institution [24]. In our setting, universal screening would be less costly than risk factor-based screening only with low MRSA prevalence (1–3%). Clancy et al. concluded that universal screening upon admission to intensive care units may be a cost avoidant strategy by decreasing healthcare-associated MRSA infections [2]. However, this study was unit-specific and compared universal screening with no screening, limiting direct comparison to our study.

Several studies have used computer simulation modelling with data derived from the literature to evaluate the costs of MRSA control measures [37]. Lee et al. utilized a computer simulation model and determined that universal MRSA admission screening would be cost-effective at prevalence rates as low as 1% [17]. Gidengil et al. conducted a microsimulation with a hypothetical cohort of adults admitted to an intensive care unit and found that, compared to admission screening or universal contact precautions, universal decolonization was the most cost-effective in preventing subsequent cases of MRSA colonization or infection [38]. Finally, McKinnell et al. used a Markov model and found no benefit to universal screening and contact precautions in preventing healthcare-associated MRSA under any conditions [39]. However, the results of these modeling studies must be interpreted with caution as they were populated with data derived from the literature. Our study had the advantage of using actual hospital-based data to compare a universal MRSA screening program with the standard risk-factor based program to provide evidence that universal screening is not cost-effective in our setting.

The outcomes from this analysis can help inform various decision-makers including infection control specialists and hospital administrators. The current results have been used to inform evidence-based policies at our hospital. As a universal MRSA admission screening program was not clinically or cost-effective in our setting, this program was discontinued [24]. Based on our analysis of patients who are at highest risk of having MRSA, our current policy includes screening all patients admitted through an emergency department, all patients admitted to an intensive care unit or the Rehabilitation Center, and direct transfers from another institution. This policy excludes elective patients admitted to an overnight stay unit, mental health patients, newborns, and obstetrical and antepartum patients. An important factor that is not explicitly factored into this analysis is the potential negative impact of contact precautions. While contact precautions are important to reduce the spread of MRSA and other infectious diseases, they can negatively affect patient mental well-being, healthcare worker attentiveness, and frequency of adverse events [40,41].

### Limitations

There are several important limitations to our study. First, because we used actual cost and probability data from our center according to best practices for economic modelling, the results are specific to our setting and may not be generalizable. For example, factors that may lower costs include laboratory costs associated with chromogenic agar (rather than PCR) for MRSA detection [42], not using multi-bed rooms (thus, avoiding costs associated with private room use), or screening the nares alone rather than multiple sites [39]. In addition, the probabilities associated with acquiring MRSA or developing an MRSA infection may be lower in centers that use routine MRSA decolonization [38]. A second potential limitation is the use of MRSA bacteremia as a measure of MRSA infection, thus excluding other potential infections (e.g. wounds, urinary tract infections, pneumonia). While this may underestimate the costs associated with MRSA infections, we chose to use bacteremias as they are the most objective measures of true MRSA infection, and associated with well-defined clinical significance. The decision to include bacteremias as our standard for MRSA infection is supported in the literature and yields a conservative, but objective, estimate of MRSA infection. Including all MRSA infections in our model would have magnified the cost differential with universal screening, but not changed our overall conclusion that universal screening is not cost-effective. A third limitation is that the model may be missing MRSA patients who were not screened or had a falsely negative screening test and are therefore ‘unknown’ MRSA positive cases. These patients would generate additional costs once their MRSA status became known (e.g. from a subsequent clinical culture). This in turn, may have falsely elevated the costs during the universal screening period as these patients were identified upon admission screening while the risk factor-based screening regimen neglected to identify similar patients and their associated costs. While we accounted for this in the sensitivity analysis, current literature is inadequate to sufficiently inform our estimates. Future population-based studies to define the probability of unscreened patients carrying MRSA on admission, and the probability of a false-negative screening test, would strengthen our sensitivity analysis. Finally, our universal admission screening intervention was not truly “universal” as only 85% of admitted patients were screened [24]. At our center, the most common reasons patients were not screened on admission included admission through our trauma emergency department directly to the operating room, patient refusal, or staff non-compliance with protocols. Nonetheless, our sensitivity analysis confirmed that improved compliance with admission screening would be associated with substantially higher costs.

## Conclusion

Despite these limitations, we believe this analysis represents an important contribution to the current literature as the first study to conduct a robust cost analysis of a hospital-wide universal MRSA admission using actual cost and probability data. These data may inform future model-based economic analyses of MRSA interventions. In our setting, a universal MRSA admission screening program cost over a million dollars per year in additional hospital costs and did not significantly affect the rates of nosocomial MRSA. However, as noted by others [7,17], more work is needed to determine whether there is a societal benefit to more universal MRSA admission screening programs, despite the additional cost to individual hospitals.

## Supporting Information

S1 Dataset MRSA costing analysis publicly available dataset. (XLS)

S1 STROBE Checklist (DOC)

S1 Table Health states and probability estimates for MRSA screening. (DOC)

S2 Table Sensitivity analysis: Cost of patient care and associated probabilities for each clinical scenario and health state including ‘false negatives’ and ‘unknown’ MRSA bacteremia cases. (DOC)
