# Supplementary material for: Cost Analysis of Universal Screening vs. Risk Factor-Based Screening for Methicillin-Resistant Staphylococcus aureus (MRSA)
Source: PLoS One. 2016 Jul 27;11(7):e0159667. doi: 10.1371/journal.pone.0159667 (PMC4963093; doi:10.1371/journal.pone.0159667)
Supplement: S1 STROBE Checklist — (DOC) [file pone.0159667.s002.doc]

S1 Checklist: STROBE checklist

|  | Item No | Recommendation |
| --- | --- | --- |
| **Title and abstract** | 1 | (*a*) Indicate the study’s design with a commonly used term in the title or the abstract  Included: Title page, abstract |
|  |  | (*b*) Provide in the abstract an informative and balanced summary of what was done and what was found  Included: Abstract, methods and results section |
| Introduction | | |
| Background/rationale | 2 | Explain the scientific background and rationale for the investigation being reported  Included: Introduction section |
| Objectives | 3 | State specific objectives, including any prespecified hypotheses  Included: Introduction section; Methods section |
| Methods | | |
| Study design | 4 | Present key elements of study design early in the paper  Included: Methods section - Study design and setting section |
| Setting | 5 | Describe the setting, locations, and relevant dates, including periods of recruitment, exposure, follow-up, and data collection  Included: Methods – Study design and setting |
| Participants | 6 | (*a*) *Cohort study*—Give the eligibility criteria, and the sources and methods of selection of participants. Describe methods of follow-up  Included: Methods – Study design and setting, Laboratory and infection control methods |
|  |  | (*b*) *Cohort study*—For matched studies, give matching criteria and number of exposed and unexposed.  This was not a matched study |
| Variables | 7 | Clearly define all outcomes, exposures, predictors, potential confounders, and effect modifiers. Give diagnostic criteria, if applicable  Included: Methods – Primary outcome, Secondary outcomes |
| Data sources/ measurement | 8* | For each variable of interest, give sources of data and details of methods of assessment (measurement). Describe comparability of assessment methods if there is more than one group  Included: Methods – Decision model section |
| Bias | 9 | Describe any efforts to address potential sources of bias  Included: Methods section – Decision model, Cost analysis, Sensitivity analysis |
| Study size | 10 | Explain how the study size was arrived at  A sample size was not calculated because the analyses used the complete administrative database. |
| Quantitative variables | 11 | Explain how quantitative variables were handled in the analyses. If applicable, describe which groupings were chosen and why  Included: Methods section – Decision model, Cost analysis, sensitivity analysis |
| Statistical methods | 12 | (*a*) Describe all statistical methods, including those used to control for confounding  Included: Methods section – Decision model, Cost analysis, Sensitivity analysis |
|  |  | (*b*) Describe any methods used to examine subgroups and interactions  Included: Methods section –Sensitivity analysis |
|  |  | (*c*) Explain how missing data were addressed  There were no missing data in this study. All information was available from the dataset. |
|  |  | (*d*) *Cohort study*—If applicable, explain how loss to follow-up was addressed  Included: Loss to follow-up was not assessed because monthly incidence was calculated |
|  |  | (*e*) Describe any sensitivity analyses  Methods section –sensitivity analysis |

Continued on next page

| Results | | |
| --- | --- | --- |
| Participants | 13* | (a) Report numbers of individuals at each stage of study—eg numbers potentially eligible, examined for eligibility, confirmed eligible, included in the study, completing follow-up, and analysed  Included: A description of the sample size is included in the methods section, a detailed description of the study sample is not included because it is described in detail in a published paper referenced in the methods section. |
|  |  | (b) Give reasons for non-participation at each stage  NA – all data was administrative and participation was not a consideration |
|  |  | (c) Consider use of a flow diagram |
| Descriptive data | 14* | (a) Give characteristics of study participants (eg demographic, clinical, social) and information on exposures and potential confounders  Included: a detailed description of the study sample is not included because it is described in detail in a published paper referenced in the methods section  24. Roth VR, Longpre T, Taljaard M, Coyle D, Suh KN, Muldoon KA, et al. Universal vs Risk Factor Screening for Methicillin-Resistant Staphylococcus aureus in a Large Multicenter Tertiary Care Facility in Canada. Infect Control Hosp Epidemiol. 2016;37: 41–48. doi:10.1017/ice.2015.230. |
|  |  | (b) Indicate number of participants with missing data for each variable of interest  Included: There were no participants with missing data in the study |
|  |  | (c) *Cohort study*—Summarise follow-up time (eg, average and total amount)  Included: there are no follow-up times for this study design. |
| Outcome data | 15* | *Cohort study*—Report numbers of outcome events or summary measures over time  Included: Results section |
|  |  |  |
|  |  |  |
| Main results | 16 | (*a*) Give unadjusted estimates and, if applicable, confounder-adjusted estimates and their precision (eg, 95% confidence interval). Make clear which confounders were adjusted for and why they were included  NA |
|  |  | (*b*) Report category boundaries when continuous variables were categorized  NA |
|  |  | (*c*) If relevant, consider translating estimates of relative risk into absolute risk for a meaningful time period  NA |
| Other analyses | 17 | Report other analyses done—eg analyses of subgroups and interactions, and sensitivity analyses  Included: Sensitivity analysis (p.8) |
| Discussion | | |
| Key results | 18 | Summarise key results with reference to study objectives  Included: Discussion section |
| Limitations | 19 | Discuss limitations of the study, taking into account sources of potential bias or imprecision. Discuss both direction and magnitude of any potential bias  Included: Discussion section |
| Interpretation | 20 | Give a cautious overall interpretation of results considering objectives, limitations, multiplicity of analyses, results from similar studies, and other relevant evidence  Included: Discussion section |
| Generalisability | 21 | Discuss the generalisability (external validity) of the study results  Included: Discussion section |
| Other information | | |
| Funding | 22 | Give the source of funding and the role of the funders for the present study and, if applicable, for the original study on which the present article is based  Included: The study received no external funding |

*Give information separately for cases and controls in case-control studies and, if applicable, for exposed and unexposed groups in cohort and cross-sectional studies.

**Note:** An Explanation and Elaboration article discusses each checklist item and gives methodological background and published examples of transparent reporting. The STROBE checklist is best used in conjunction with this article (freely available on the Web sites of PLoS Medicine at http://www.plosmedicine.org/, Annals of Internal Medicine at http://www.annals.org/, and Epidemiology at http://www.epidem.com/). Information on the STROBE Initiative is available at www.strobe-statement.org.
