# Supplementary material for: Cost Analysis of Universal Screening vs. Risk Factor-Based Screening for Methicillin-Resistant Staphylococcus aureus (MRSA)
Source: PLoS One. 2016 Jul 27;11(7):e0159667. doi: 10.1371/journal.pone.0159667 (PMC4963093; doi:10.1371/journal.pone.0159667)
Supplement: S1 Table — (DOC) [file pone.0159667.s003.doc]

S1 Table : Health states and probability estimates for MRSA screening

| **Health State** | **Parameter description** | **Probability estimate** | **Associated costs** | **Source** | **Node** |
| --- | --- | --- | --- | --- | --- |
| Screened |  |  |  |  |  |
| Risk factor-based screening | Screened for MRSA within 48h of admission | 0.292 | Yes | Actual data | P1 |
| Universal screening | Screened for MRSA within 48h of admission | 0.838 | Yes | Actual data | P1 |
| Not screened | Not screened for MRSA within 48h of admission |  |  |  |  |
| Risk factor-based screening | Screened for MRSA within 48h of admission | 0.708 | No | Actual data | P2 |
| Universal screening | Screened for MRSA within 48h of admission | 0.162 | No | Actual data | P2 |
| Negative PCR | Admission screen for MRSA is PCR negative | 0.955 | Yes | Actual data | P3 |
| Positive PCR | Admission screen for MRSA is PCR positive | 0.045 | Yes | Actual data | P4 |
| Negative culture | PCR positive specimen is MRSA culture negative (false positive PCR) | 0.350 | Yes | Actual data | P21 |
| Positive culture | PCR positive specimen is MRSA culture positive (true positive PCR) | 0.650 | Yes | Actual data | P20 |
| MRSA negative | MRSA negative, but not screened on admission | 0.952 | No | Derived from Conterno et al.^26^ | P5 |
| Known MRSA positive | Identified as MRSA positive on previous admission | 0.030 | Yes | Conterno et al.^26^ | P6 |
| Unknown MRSA positive | MRSA status unknown | 0.018 | No | Derived from Conterno et al.^26^ | P7 |
| True negative | Patient is MRSA negative based on negative predictive value of PCR test (true negative PCR) | 0.980 | No | Conterno et al.^26^ | P8 |
| False negative | Patient is MRSA positive despite PCR negative result, based on negative predictive value of PCR test (false negative PCR) | 0.020 | No | Conterno et al.^26^ | P9 |
| Does not acquire MRSA | Remains MRSA negative during hospital admission | 0.995 | No | Actual data | P10/P24 |
| Acquires MRSA | MRSA negative upon hospital admission but acquired MRSA during hospital stay | 0.005 | Yes | Actual data | P11/P25 |
| Bacteremia | Patient tests positive for MRSA bacteremia | 0.043 | Yes | Actual data | P15/P26/P32 |
| Colonized | Colonized with MRSA | 0.957 | Yes | Actual data | P14/P27/P33 |
| Death |  |  |  |  |  |
| Death – MRSA bacteremia | MRSA bacteremia patient dies while in hospital | 0.130 | No | Coello et al.^29^ | P16/P28/P34 |
| Death – MRSA colonized | MRSA colonized patient dies while in hospital | 0.033 | No | Van Walraven et al.^28^ | P18/P23/P30/P36 |
| Death – MRSA negative | MRSA negative patient dies while in hospital | 0.033 | No | Van Walraven et al.^28^ | P13 |
| Discharged | Patient is discharged from hospital | 0.967 | No | Derived based on probability of death | P12/P17/P19/P22/  P29/P31/P35/P37 |
|  | | | | | |
