# Supplementary material for: Cost Analysis of Universal Screening vs. Risk Factor-Based Screening for Methicillin-Resistant Staphylococcus aureus (MRSA)
Source: PLoS One. 2016 Jul 27;11(7):e0159667. doi: 10.1371/journal.pone.0159667 (PMC4963093; doi:10.1371/journal.pone.0159667)
Supplement: S2 Table — (DOC) [file pone.0159667.s004.doc]

S2 Table: Sensitivity analysis: Cost of patient care and associated probabilities for each clinical scenario and health state including ‘false negatives’ and ‘unknown’ MRSA bacteremia cases.

| **State** | **Cost / pt** | **Risk factor-based screening probability** | **Universal screening probability** | **Risk factor-based screening cost** | **Universal screening cost** | **Reference to model** |
| --- | --- | --- | --- | --- | --- | --- |
| Screened for MRSA – PCR neg. – True neg. – No acquisition – Discharged | $19.92 | 0.261142165 | 0.756411788 | $5.20 | $15.07 | P12 |
| Screened for MRSA – PCR neg. – True neg. – No acquisition - Death | $19.92 | 0.00891178 | 0.025813432 | $0.18 | $0.51 | P13 |
| **Screened for MRSA – PCR neg. - False neg. - Bacteremia - Death** | **$25,932.77** | **3.0963E-05** | **8.9686E-05** | **$0.80** | **$2.33** | **P16** |
| **Screened for MRSA – PCR neg. - False neg. - Bacteremia - Discharge** | **$25,932.77** | **0.000207214** | **0.000600206** | **$5.37** | **$15.57** | **P17** |
| Screened for MRSA – PCR neg. - False neg. - Colonized - Death | $19.92 | 0.000174927 | 0.000506686 | $0.00 | $0.01 | P18 |
| Screened for MRSA – PCR neg. - False neg. - Colonized - Discharge | $19.92 | 0.005125896 | 0.014847422 | $0.10 | $0.30 | P19 |
| Screened for MRSA - PCR pos. – Culture neg. - Discharge | $811.97 | 0.004416773 | 0.01279341 | $3.59 | $10.39 | P22 |
| Screened for MRSA - PCR pos. – Culture neg. - Death | $811.97 | 0.000150728 | 0.00043659 | $0.12 | $0.35 | P23 |
| Not screened for MRSA – MRSA neg. - No acquisition - Discharge | $0.00 | 0.650073312 | 0.146495394 | $0.00 | $0.00 | P22 |
| Not screened for MRSA– MRSA neg. - No acquisition - Death | $0.00 | 0.022184508 | 0.004999326 | $0.00 | $0.00 | P23 |
| **Not screened for MRSA - Unknown MRSA status - Bacteremia - Death** | **$25,929.44** | **7.30278E-05** | **1.6457E-05** | **$1.89** | **$0.43** | **P28** |
| **Not screened for MRSA - Unknown MRSA status - Bacteremia - Discharge** | **$25,929.44** | **0.000488724** | **0.000110135** | **$12.67** | **$2.86** | **P29** |
| Not screened for MRSA - Unknown MRSA status - Colonized - Death | $0.00 | 0.000412574 | 9.29745E-05 | $0.00 | $0.00 | P30 |
| Not screened for MRSA - Unknown MRSA status - Colonized - Discharge | $0.00 | 0.012089674 | 0.002724434 | $0.00 | $0.00 | P31 |
| Screened for MRSA – PCR neg. - True neg. - Acquire MRSA - Bacteremia - Death | $25,949.36 | 7.58594E-06 | 2.19731E-05 | $0.20 | $0.57 | P34 |
| Screened for MRSA – PCR neg.- True neg. - Acquire MRSA - Bacteremia - Discharge | $25,949.36 | 5.07674E-05 | 0.00014705 | $1.32 | $3.82 | P35 |
| Screened for MRSA – PCR neg. - True neg. - Acquire MRSA - Colonized - Death | $2,616.42 | 4.28572E-05 | 0.000124138 | $0.11 | $0.32 | P36 |
| Screened for MRSA – PCR neg. - True neg. - Acquire MRSA - Colonized - Discharge | $2,616.42 | 0.001255844 | 0.003637618 | $3.29 | $9.52 | P37 |
| Screened for MRSA – PCR pos. – Culture pos. - Bacteremia - Death | $25,819.46 | 4.74172E-05 | 0.000137346 | $1.22 | $3.55 | P34 |
| Screened for MRSA – PCR pos. – Culture pos. - Bacteremia - Discharge | $25,819.46 | 0.00031733 | 0.000919164 | $8.19 | $23.73 | P35 |
| Screened for MRSA – PCR pos. – Culture pos. - Colonized - Death | $2,494.26 | 0.000267886 | 0.000775945 | $0.67 | $1.94 | P36 |
| Screened for MRSA – PCR pos. – Culture pos. - Colonized – Discharged | $2,494.26 | 0.007849867 | 0.022737545 | $19.58 | $56.71 | P37 |
| Not screened for MRSA- MRSA neg. - Acquire MRSA- Bacteremia - Death | $25,929.44 | 1.8884E-05 | 4.25556E-06 | $0.49 | $0.11 | P34 |
| Not screened for MRSA – MRSA neg. - Acquire MRSA - Bacteremia - Discharge | $25,929.44 | 0.000126378 | 2.84795E-05 | $3.28 | $0.74 | P35 |
| Not screened for MRSA – MRSA neg. - Acquire MRSA - Colonized - Death | $2,596.50 | 0.000106686 | 2.4042E-05 | $0.28 | $0.06 | P36 |
| Not screened for MRSA – MRSA neg. - Acquire MRSA - Colonized - Discharge | $2,596.50 | 0.003126232 | 0.000704503 | $8.12 | $1.83 | P37 |
| Not screened for MRSA - Known MRSA pos. - Bacteremia - Death | $25,698.51 | 0.000119067 | 0.000026832 | $3.06 | $0.69 | P34 |
| Not screened for MRSA - Known MRSA pos. - Bacteremia - Discharge | $25,698.51 | 0.000796833 | 0.000179568 | $20.48 | $4.61 | P35 |
| Not screened for MRSA - Known MRSA pos. - Colonized - Death | $2,382.16 | 0.00067078 | 0.000152536 | $1.60 | $0.36 | P36 |
| Not screened for MRSA - Known MRSA pos. - Colonized - Discharge | $2,382.16 | 0.019711425 | 0.004442011 | $46.96 | $10.58 | P37 |
| **TOTAL** |  | **1** | **1** | **$148.77** | **$166.95** | **(-$18.18)** |
